# Piroplasmosis in wildlife: *Babesia* and *Theileria* affecting free-ranging ungulates and carnivores in the Italian Alps

**DOI:** 10.1186/1756-3305-7-70

**Published:** 2014-02-17

**Authors:** Stefania Zanet, Anna Trisciuoglio, Elisa Bottero, Isabel Garcia Fernández de Mera, Christian Gortazar, Maria Grazia Carpignano, Ezio Ferroglio

**Affiliations:** 1 Department of Veterinary Sciences, University of Turin, via Leonardo da Vinci, 44, 10095 Grugliasco, TO, Italy; 2 SaBio IREC CSIC UCLM JCCM, Ronda de Toledo s/n. 13071, Ciudad Real, Spain; 3 Comprensorio Alpino Valli Grana e Maira, via Roma 28, 12025 Dronero, CN, Italy

**Keywords:** Piroplasmosis, *Babesia*, *Theileria*, PCR, Wildlife, Italy

## Abstract

**Background:**

Piroplasmosis are among the most relevant diseases of domestic animals. *Babesia* is emerging as cause of tick-borne zoonosis worldwide and free-living animals are reservoir hosts of several zoonotic *Babesia* species. We investigated the epidemiology of *Babesia* spp. and *Theileria* spp. in wild ungulates and carnivores from Northern Italy to determine which of these apicomplexan species circulate in wildlife and their prevalence of infection.

**Methods:**

PCR amplification of the V4 hyper-variable region of the 18S rDNA of *Babesia* sp./*Theileria* sp was carried out on spleen samples of 1036 wild animals: Roe deer *Capreolus capreolus* (n = 462), Red deer *Cervus elaphus* (n = 52)*,* Alpine Chamois *Rupicapra rupicapra* (n = 36)*,* Fallow deer *Dama dama* (n = 17), Wild boar *Sus scrofa* (n = 257), Red fox *Vulpes vulpes* (n = 205) and Wolf *Canis lupus* (n = 7). Selected positive samples were sequenced to determine the species of amplified *Babesia*/*Theileria* DNA.

**Results:**

*Babesia*/*Theileria* DNA was found with a mean prevalence of 9.94% (IC95% 8.27-11.91). The only piroplasms found in carnivores was *Theileria annae*, which was detected in two foxes (0.98%; IC95% 0.27-3.49). Red deer showed the highest prevalence of infection (44.23%; IC95% 31.6-57.66), followed by Alpine chamois (22.22%; IC95% 11.71-38.08), Roe deer (12.55%; IC95% 9.84-15.89), and Wild boar (4.67%; IC95% 2.69-7.98). Genetic analysis identified *Babesia capreoli* as the most prevalent piroplasmid found in Alpine chamois, Roe deer and Red deer, followed by *Babesia bigemina* (found in Roe deer, Red deer and Wild boar), and the zoonotic *Babesia venatorum* (formerly *Babesia* sp. EU1) isolated from 2 Roe deer. Piroplasmids of the genus *Theileria* were identified in Wild boar and Red deer.

**Conclusions:**

The present study offers novel insights into the role of wildlife in *Babesia*/*Theileria* epidemiology, as well as relevant information on genetic variability of piroplasmids infecting wild ungulates and carnivores.

## Background

*Babesia* spp. and *Theileria* spp. are protozoan parasites transmitted mainly by ticks and able to infect erythrocytes and/or leukocytes of a wide variety of domestic and wild animals [1] and *Babesia* is the second most common parasite found in the blood of mammals after trypanosomes [2]. Some species of the genus *Theileria* such as *T. annulata* and *T. parva* are highly pathogenic to cattle and cause significant mortality among susceptible animals [3,4]. Other *Theileria* spp. are considered to be benign or less pathogenic probably because of a long evolutionary relationship between the parasite and the host. Nevertheless, clinical disease may occur in stressful situations related to translocation of wildlife or/and when the host is debilitated by other parasitic organisms or malnutrition [5-7]. Although piroplasmosis is among the most relevant diseases of domestic animals [8,9], many unknowns remain concerning their epidemiology and life cycle in the Ixodid tick vector as well as in the vertebrate host, especially for factors involved in the role of wildlife as reservoirs of infection [2,10]. Recently, white-tailed deer were indicated as being responsible for reintroducing the tick vector of Cattle Tick Fever in Central Texas and hence the piroplasms responsible for bovine babesiosis (*Babesia bovis* and *Babesia bigemina*) [11]. Different *Theileria* and *Babesia* species were reported in wildlife with high prevalence of infection [5,9,12], some of which are recognized zoonotic pathogens, such as *Babesia divergens*[13], *Babesia divergens*-like [14,15], *Babesia venatorum* (formerly *Babesia* sp. EU1) [16-18], *Babesia microti*[2,19,20] and *B. microti*-like [21,22]. No *Theileria* species have to date been identified as causative agents of zoonotic infections [2]. The epidemiology of *Babesia* and *Theileria* in European wildlife is complex. Besides the variety of host species susceptible to infection (i.e. cervids: Roe deer *Capreolus capreolus*, Red deer *Cervus elaphus*, Fallow deer *Dama dama*; bovids: Alpine Chamois *Rupicapra rupicapra,* Spanish Ibex *Capra pyrenaica*; suids: wild boar *Sus scrofa*) [5,23-25], different tick vectors contribute to piroplasmid transmission: *Ixodes ricinus* is the most common vector of *Babesia* species in Europe but recently, 5 species of ticks were found to be infected with piroplasmids in Central and Northern Italy [26], suggesting the need to investigate other potential vector species since novel tick/pathogen associations were detected in *Hyalomma marginatum*, *Rhipicephalus sanguineus* and *I. ricinus*[26]. In North America, *I. scapularis* is the primary vector responsible for transmission of *B. microti* to humans [2]. As for *Babesia*, the occurrence of *Theileria* depends on the simultaneous presence of appropriate vector ticks and host species. In the USA, the lone-star tick *Ambloymma americanum* is the only known vector of *T. cervi*, which mainly infects wild ruminants, particularly White-tailed deer [27,28]. In Europe, little is known about tick species involved in the transmission of *Theileria* to cervids, and it is assumed that ticks of the genera *Ixodes*, *Hyalomma* and *Rhiphicephalus* might be involved [5,7]. As biomolecular tools are easily available, in recent years many studies have investigated the epizootiology of piroplasmids circulating in wildlife, leading to the identification of several *Babesia* and *Theileria* species strictly related to wild animals or of strains shared with domestic animals [11,29]. The novel *B. venatorum* is strictly related to Roe deer presence [24], while *B. capreoli* was detected in Red deer from Ireland, where Roe deer are absent [24]. *C. capreolus* was also identified as a possible source of fatal babesial infection for the Alpine chamois [30,31]. The territorial expansion and population increase that involved Roe deer in several European countries including Italy [32], led to increased contacts and spatial overlap with *R. rupicapra* populations and hence to an increased possibility of infection of this species with piroplasms of cervids such as *B. capreoli*[23]. The zoonotic *B. divergens* is also reported to infect European wild ungulates and has probably one of the largest host ranges described to date for a *Babesia* species [33], although Malandrin *et al*. [13] recently reviewed previous identifications of this parasite attributing many of them to *B. capreoli*. In contrast to ungulates, information on the occurrence and prevalence of piroplasmids in wild canids is limited [34]. In Europe, a continent where the Red fox is present at high densities [35], *B. microti*-like piroplasms were molecularly confirmed in foxes from Central and Northern Spain [9,36,37], Croatia [38] and Portugal [34]. In Portugal, a single fox was also found infected with *B. canis*[34]. Considering the relevant insights into piroplasm-wildlife epidemiology [39,40], the increasing importance of *Babesia* as an emerging zoonotic disease [2,10] and the lack of information on piroplasm epidemiology in Northwestern Italy [23], we widely investigated *Babesia*/*Theileria* infection prevalence in wild ungulates and carnivores from the Piedmont region (Italy) and their biomolecular characteristics, by amplifying and sequencing part of the V4 hyper-variable region of the 18S rRNA gene.

## Methods

A total of 1036 free-ranging wild ungulates and carnivores were sampled during a period of 5 years (from 2008 to 2012), in a wide area of Northern Italy (2 million hectares), that includes a wide range of habitats and different ecological niches ranging from low altitude intensively cultivated farm land to Alpine forests and meadows. The animals included in the study were either culled by hunters, accidentally found dead, or culled within official programs for species demographic control. Ethical and institutional approval was given by the Department of Veterinary Sciences, University of Turin (Italy). Six species of ungulates were sampled: Roe deer *Capreolus capreolus* (n = 462), Red deer *Cervus elaphus* (n = 52)*,* Alpine chamois *Rupicapra rupicapra* (n = 36)*,* Fallow deer *Dama dama* (n = 17) and Wild boar *Sus scrofa* (n = 257). Two carnivore species, Red fox *Vulpes vulpes* (n = 205) and Wolf *Canis lupus* (n = 7) were also included in the study. All animals were promptly brought to the Turin Veterinary Faculty for necropsy where a portion of splenic tissue was collected from each animal and stored at -20°C until further processing. All standard precautions were taken to minimize the risk of cross-contamination (preparation of PCR and addition of DNA was carried out in separate laminar-flow cabinets using DNA-free disposable material. Positive and negative control samples were processed in parallel with all samples). Total genomic DNA was extracted from ≈ 10 mg of spleen using the commercial kit GenElute® Mammalian Genomic DNA Miniprep (Sigma–Aldrich, St. Louis, MO, USA) according to manufacturer’s instructions. Direct molecular detection of *Babesia* spp./*Theileria* spp. DNA was carried out using a semi-nested PCR protocol targeting the V4 hyper-variable region of the 18S ribosomal RNA gene. The primers used are highly conserved and can amplify a wide variety of *Babesia* and *Theileria* species [41,42]. The first amplification was carried out using the primers RLB-F2 (5’-GACACAGGGAGGTAGTGACAAG-3’) and RLB-R2 (5’-CTAAGAATTTCACCTCTGACAGT-3’) [41]. The PCR reaction was carried out in a final volume of 25 μl, using Promega PCR Master Mix (Promega Corporation, WI, USA), 20pM of each primer, and ≈ 100 ng of DNA template. The amplification included a 5 min denaturation step at 95°C followed by 25 repeats of 30 s at 95°C, 45 s at 50°C, and 1.5 min at 72°C and a final extension at 72°C for 10 min. Amplicons (1 μl) of the first PCR step were used as template for the second amplification which used RLB-FINT (5’-GACAAGAAATAACAATACRGGGC-3’) [43] as internal forward primer, together with RLB-R2. The reaction mixture and cycling program were identical to the direct RLB-F2/RLB-R2 amplification, but the cycle number was increased to 40, whereas the annealing temperature was 50°C in the first PCR and 55°C in the second PCR. A positive and a negative control sample were included in each amplification reaction. Amplicons were analyzed by agarose gel electrophoresis (2%) and visualized by staining with Gel Red Nucleic Acid Gel Stain (VWR International Milano, Italy). Selected positive amplicons were purified (QIAquick PCR purification kit, QIAGEN) and both DNA strands were directly sequenced (Macrogen; http://www.macrogen.com). The resulting sequences were compared with the NCBI/Genbank database using the Basic Local Alignment Search Tool (BLAST), and ClustalX software (http://www-igbmc.u-strasbg.fr/BioInfo) was used to construct multiple-sequence alignments. All statistical analysis was carried out using R software 3.0.1 (R Core Team 2012). Chi-square test was used to assess potential differences in *Babesia*/*Theileria* infection rate between groups (animals were grouped by species, age, sex, and year of sampling).

## Results

### Prevalence of *Babesia/Theileria* spp. DNA in wildlife

Piroplasm DNA was detected with an overall prevalence of 9.94% (IC95% 8.27-11.91). Detailed prevalence data for each species are reported in Table 1. Herbivores (P = 15.7%; IC95% 12.93-18.92) were significantly more infected (χ^2^ = 32.55, p < 0.0000; OR = 19.55, 4.77-80.14) than carnivores (P = 0.94%; IC95% 0.26-3.37) as *Babesia*/*Theileria* DNA was amplified from two foxes, while all the results from the wolves examined were negative by PCR. Among herbivores, the highest prevalence of infection was reported in Red deer (P = 44.23%; IC95% 31.6-57.66). This species was significantly more infected than the other examined ungulates (χ^2^ = 51.06, p < 0.0000; OR = 6.88, 3.8-12.48). On the other hand, Wild boar results showed significantly less infection than herbivore species (χ^2^ = 20.00, p < 0.0000; OR = 0.2631, 0.14-0.49). Sex and age did not significantly influence the infection status of any of the species tested (data not shown), nor any statistically significant difference between sampling years was recorded (data not shown).

**Table 1:** **Prevalence of *Babesia*/*Theileria *** **spp. in wildlife species**

**Species**	**Prevalence (IC95%)**	**Positive/total sampled**
Roe deer	12.55% [9.84-15.89]	58/462
Wild boar	4.67% [2.69/7.98]	12/257
Fallow deer	0.00% [0.00/18.43]	0/17
Alpine chamois	22.22% [11.71/38.08]	8/36
Red deer	44.23% [31.6/57.66]	23/52
Red fox	0.98% [0.27/3.49]	2/205
Grey Wolf	0.00% [0.00/35.43]	0/7
**Total**	**9.94% [103/1036]**	**103/1036**

PCR prevalence to *Babesia*/*Theileria* differs greatly among sampled species. We reported detailed prevalence values and confidence intervals (95%), together with the number of tested and positive animals of each sampled species.

### Sequencing and molecular classification

**Figure 1:**
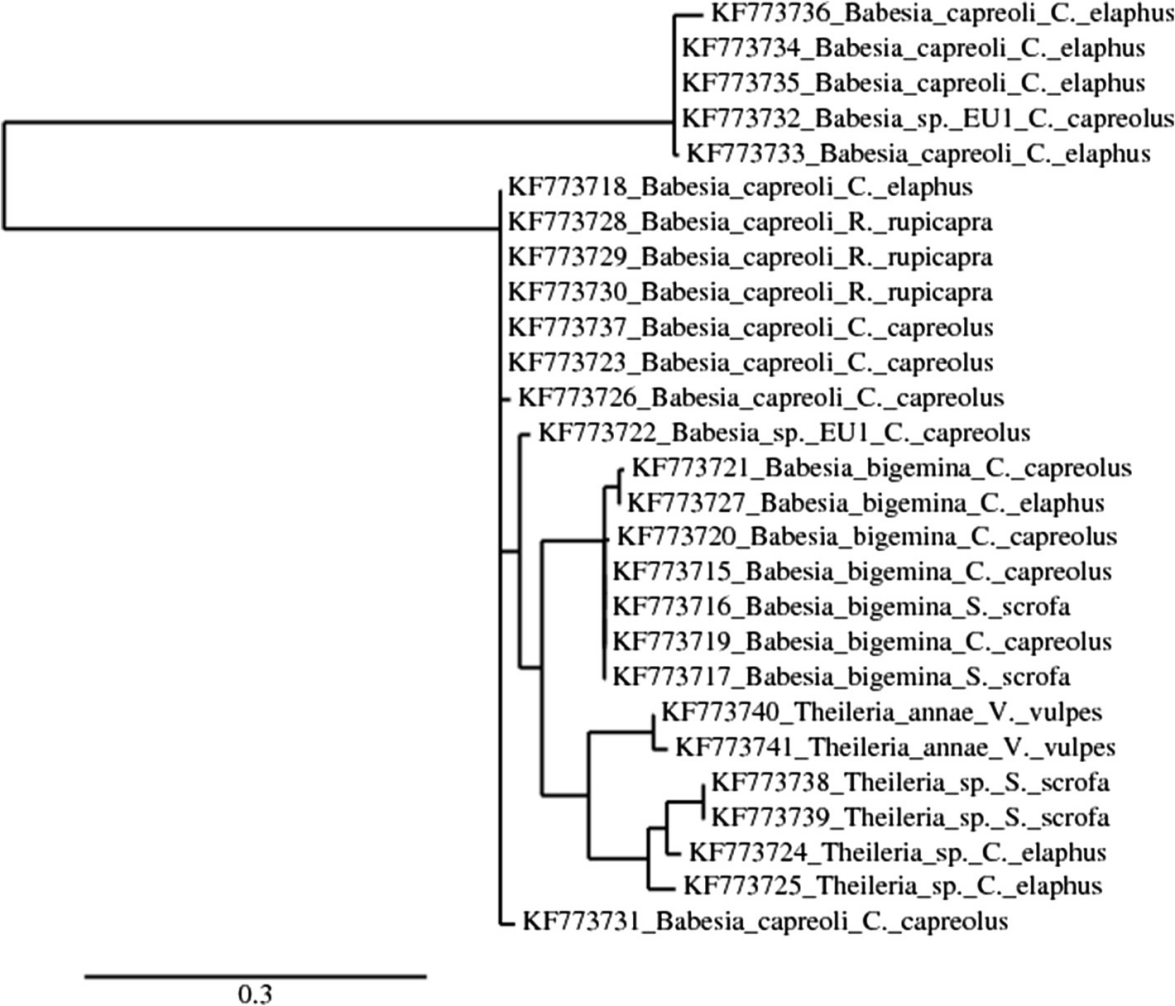
**Phylogenetic tree of the *Babesia/Theileria *isolates from carnivores and ruminants of Northwestern Italy performed using Neighbor-joining and 1000 bootstrap replicates.** The samples were coded with their corresponding GenBank accession number.

A total of 28 positive samples were sequenced and deposited in GenBank under accession numbers from KF773715 to KF773741 (Table 2)*. B. capreoli* was the most prevalent species (P = 46.43%; CI95% 29.53-64.19), it was found in Alpine chamois (n = 4), Roe deer (n = 4), and Red deer (n = 5). All four *B. capreoli* isolates from Alpine chamois [GenBank: from KF773728 to KF773730] showed 100% identity with the *B. capreoli* described by Hoby *et al*., [30] in fatal cases of babesiosis in chamois from Switzerland [GenBank:EF545558 to EF545562], while a Red deer isolate [Genbank: KF773718] showed 100% homology with *B. capreoli* [GenBank: FJ944827- FJ944828] identified in Roe deer in France [13]. *B. bigemina* was the second most prevalent *Babesia* species. It was identified in Roe deer (n = 4), Red deer (n = 1) and Wild boar (n = 2). All isolates showed 99% identity with *B. bigemina* [Genbank: HQ264116], infecting White-tailed deer in Southern Texas [11]. Roe deer (n = 2) were also infected with the zoonotic *B. venatorum*. Both isolates showed 100% identity with the *B. venatorum* [GenBank: AY046575] described in two human cases in Italy and Austria [16]. Piroplasms of the genus *Theileria* were detected in Wild boar (n = 2) and Red deer (n = 2). Wild boar isolates showed 100% identity with *Theileria* sp. CS-2012 [Genbank: JQ751279] described in Wild boars and Sambar deer in Thailand. An isolate from Red deer [Genbank: KF773724] showed 100% identity with *Theileria* sp. 3185/02 described in a Red deer imported from Germany to Spain [Genbank: AY421708] [7], while another Red deer isolate [GenBank: KF773725] showed 100% identity with *Theileria* sp. OT3 described in chamois, deer and sheep from Spain [GenBank: DQ866840] [44,45]. *T. annae* (syn. *B. microti*-like) was detected in the two PCR positive foxes. One isolate [GenBank: KF773740] showed 100% identity with *T. annae* amplified from Croatian foxes [GenBank: HM212628] [38], while the second isolate [Genbank: KF773741], although very similar, has CT instead of AA at 268–269 pb. The phylogenetic relationships among the sequenced samples are reported in Figure 1.

**Table 2:** ***Babesia*/*Theileria *** **species identification**

**Host**	**Area of origin**	***Babesia/Theileria *** **specie**	**GenBank accession no.**
Chamois [*R. rupicapra*]	SW Piedmont, Cuneo Province	*B. capreoli*	KF773728*
	SW Piedmont, Cuneo Province	*B. capreoli*	KF773729
	SW Piedmont, Cuneo Province	*B. capreoli*	KF773730
	NW Piedmont, Torino Province	*B. capreoli*	KF773728*
Red deer [*C. elaphus*]	NW Piedmont, Torino Province	*B. capreoli*	KF773718
	NW Piedmont, Torino Province	*Theileria sp.*	KF773724
	SE Piedmont, Alessandria Province	*Theileria sp.*	KF773725
	NW Piedmont, Torino Province	*B. bigemina*	KF773727
	NW Piedmont, Torino Province	*B. capreoli*	KF773733
	SW Piedmont, Cuneo Province	*B. capreoli*	KF773734
	NE Piedmont, Biella Province	*B. capreoli*	KF773735
	SE Piedmont, Alessandria Province	*B. capreoli*	KF773736
Roe deer [*C. capreolus*]	SE Piedmont, Alessandria Province	*B. bigemina*	KF773715
	NW Piedmont, Torino Province	*B. bigemina*	KF773719
	SW Piedmont, Cuneo Province	*B. bigemina*	KF773720
	SW Piedmont, Cuneo Province	*B. bigemina*	KF773721
	NW Piedmont, Torino Province	*B. venatorum*	KF773722
	NW Piedmont, Torino Province	*B. capreoli*	KF773723
	SE Piedmont, Alessandria Province	*B. capreoli*	KF773724
	SE Piedmont, Alessandria Province	*B. capreoli*	KF773731
	NW Piedmont, Torino Province	*B. venatorum*	KF773732
	SW Piedmont, Cuneo Province	*B. capreoli*	KF773737
Wild boar [*S. scrofa*]	SE Piedmont, Alessandria Province	*B. bigemina*	KF773716
	NW Piedmont, Torino Province	*B. bigemina*	KF773717
	SE Piedmont, Alessandria Province	*Theileria sp.*	KF773738
	SE Piedmont, Alessandria Province	*Theileria sp.*	KF773739
Red fox [*V. vulpes*]	NW Piedmont, Torino Province	*T. annae*	KF773740
	NW Piedmont, Torino Province	*T. annae*	KF773741

*Babesia*/*Theileria* species was determined, for selected positive samples, by sequencing of PCR products. For each of the five animal species in which piroplasmid DNA was detected we reported the parasite species, the GenBank accession number attributed to each isolate and the area of origin. *A unique accession number was attributed to two chamois.

## Discussion

Piroplasmids are frequently found to infect free-living animals worldwide and are gaining increasing attention as emerging tick-borne zoonosis [16,46,47]. Increased wildlife-human interactions due to socio-economic changes have enhanced the risk of contracting zoonotic diseases [23]. This is especially true for vector-borne pathogens where the presence of the vector in the area has increased thanks to climatic changes and/or changes in the environment or in host species presence [48-50]. *Babesia* and *Theileria* are traditionally known for their relevant economic impact on the livestock industry and on human health. Several wild animals are known to be reservoirs of zoonotic *Babesia* species [2]. Although piroplasmosis in wildlife is mostly asymptomatic [46] recent cases of fatal babesiosis were recorded in Alpine chamois infected with *B. capreoli*[30,31]. Malandrin *et al.*, [13] clarified the ambiguous classification of *B. divergens*-like parasites isolated from cervids, revealing that most infections reported from wildlife can likely be ascribed to *B. capreoli*. Amplification and sequencing of the 18S rRNA gene was essential to correctly identify the two species (*B. divergens* and *B. capreoli*) as distinct. PCR protocols targeting the V4 hyper-variable region of 18S rDNA, like the one used in the present study, are able to identify a wide variety of *Babesia*/*Theileria* species and may also detect less closely related genotypes, such as the *B. microti* complex or species related to *B. odocoilei*[29]. In our study no *B. divergens* was identified in any of the sequenced samples, while the closely related *B. capreoli* was the most prevalent species, confirming that where Roe deer are abundant, *B. capreoli* is also widely present. *B. venatorum* was first identified in splenectomized human patients in Italy and Austria [16] and in Roe deer from Eastern Italy [23,51], but to our knowledge this is the first report of *B. venatorum* from the Western Alps. The high homology found between the 18S rDNA sequences of *B. venatorum* deposited in Genbank suggests that the parasite widely circulates among wild ungulates across Europe. Over the past 30 years, reintroduction of both Red deer and Roe deer occurred for restocking purposes from Central Europe and from the Balkans to the studied area [52] and the founding effect of animal translocation could also be implied for the high homology of *B. venatorum* found across the Alps. In consideration of *B. bigemina*, in the current study, it was detected in Roe deer, Red deer and Wild boar. Even if *B. bovis* and *B. bigemina* infection have been already reported in White-tailed deer from North America [11], to our knowledge this is the first report of *B. bigemina* infecting wild ungulates in Europe, suggesting the existence of a common epidemiological cycle among wildlife and sympatric livestock, since cattle are the recognized reservoir of this parasite [11,53]. The prevalence of infection recorded in our study differs greatly between the species considered. Red deer showed the highest prevalence of infection with 44% of sampled Red deer positive by PCR. Prevalences reported for the same species in Italy (12.5%) [23] as well as in other countries are significantly lower (in Ireland 26%) [24], in USA 12% [11]. Many Red deer populations present in Piedmont are characterized by high densities of animals [54]. In particular, 62.5% of *Babesia*-infected Red deer came from a low altitude fenced forest (Parco Regionale La Mandria; 3600 ha) mainly composed of broad-leaved trees and permanent meadows where the estimated Red deer population is very high (6–14 heads/km^2^) [55]. Tick-favorable conditions together with the high density population and gregarious habits of *C. elaphus* are possible causes of the high infection rate we found in Red deer. The Roe deer, even if less gregarious and territorial [56], is the second most infected species in Piedmont, confirming the high susceptibility of *C. capreolus* to piroplasmid infection. The prevalence of *Babesia*/*Theileria* DNA we found in Piedmont (12.55%) falls within the range reported for the species in Italy and in other European countries [17,23,29]. Our data also confirmed the low susceptibility of wild boar which were found infected at low prevalence (P = 4.67%), as reported also from Eastern Italy (2.6%) [23]. *T. annae* is the only piroplasmid we detected in Red foxes. Natural infection of *V. vulpes* with *T. annae* had already been documented with the prevalence of infection being higher than recorded in the Western Alps, in several European countries including Italy (50% [1/2]) [23], Northern Spain (20% [1/5]) [37], Portugal (69.2% [63/91] [34] and Croatia (5.2% [10/191]) [38].

## Conclusions

The role of wildlife as reservoirs of zoonotic *Babesia* species, as well as the involvement of free-ranging ungulates in the epidemiology of piroplasmids of veterinary importance is well established [2,39,57]. Nevertheless, many gaps in host-piroplasmid and host-tick interactions remain. For many of the numerous *Babesia*/*Theileria* species, some relevant biological aspects (e.g. reservoir hosts(s), and vectors(s)) as well as molecular characteristics remain poorly investigated. Yabsley *et al*., [2] reported how PCR-based studies with sequence analysis of piroplasms circulating in wildlife are needed to identify the causative agents of novel cases of human babesiosis as well as for managing effective surveillance plans on potential reservoirs and vectors. The data presented in this study give valuable insights on the piroplasms circulating in free-ranging ungulates and carnivores in an extensive area where wildlife lives in sympatry with a high-density human and livestock population.

## Competing interests

The authors declare that they have no competing interests.

## Authors’ contributions

SZ carried out the molecular studies with EB, participated in the sequence analysis and wrote the first draft of the manuscript; AT participated and supervised the laboratory work, MGC directly provided many of the tested chamois samples and organized sample collection in the field, IGFDM and CG collaborated to PCR setup and sample sequencing, EF coordinated the investigation, and finalized the manuscript. All authors read and approved the final manuscript.
